# Near-Infrared Cerebrovascular Reactivity for Monitoring Cerebral Autoregulation and Predicting Outcomes in Moderate to Severe Traumatic Brain Injury: Proposal for a Pilot Observational Study

**DOI:** 10.2196/18740

**Published:** 2020-08-12

**Authors:** Alwyn Gomez, Joshua Dian, Logan Froese, Frederick Adam Zeiler

**Affiliations:** 1 Section of Neurosurgery, Department of Surgery Rady Faculty of Health Sciences University of Manitoba Winnipeg, MB Canada; 2 Biomedical Engineering Faculty of Engineering University of Manitoba Winnipeg, MB Canada; 3 Department of Anatomy and Cell Science Rady Faculty of Health Sciences University of Manitoba Winnipeg, MB Canada; 4 Centre on Aging University of Mantioba Winnipeg, MB Canada; 5 Division of Anaesthesia Department of Medicine University of Cambridge Cambridge United Kingdom

**Keywords:** autoregulation, cerebrovascular reactivity, near-infrared spectroscopy, non-invasive, neurology, brain injury, trauma, protocol, outcome, invasive

## Abstract

**Background:**

Impaired cerebrovascular reactivity after traumatic brain injury (TBI) in adults is emerging as an important prognostic factor, with strong independent association with 6-month outcomes. To date, it is unknown if impaired cerebrovascular reactivity during the acute phase is associated with ongoing impaired continuously measured cerebrovascular reactivity in the long-term, and if such measures are associated with clinical phenotype at those points in time.

**Objective:**

We describe a prospective pilot study to assess the use of near-infrared spectroscopy (NIRS) to derive continuous measures of cerebrovascular reactivity during the acute and long-term phases of TBI in adults.

**Methods:**

Over 2 years, we will recruit up to 80 adults with moderate/severe TBI admitted to the intensive care unit (ICU) with invasive intracranial pressure (ICP) monitoring. These patients will undergo high-frequency data capture of ICP, arterial blood pressure (ABP), and NIRS for the first 5 days of care. Patients will then have 30 minutes of noninvasive NIRS and ABP monitoring in the clinic at 3, 6, and 12 months post-injury. Outcomes will be assessed via the Glasgow Outcome Scale and Short Form-12 questionnaires. Various relationships between NIRS and ICP-derived cerebrovascular reactivity metrics and associated outcomes will be assessed using biomedical signal processing techniques and both multivariate and time-series statistical methodologies.

**Results:**

Study recruitment began at the end of February 2020, with data collection ongoing and three patients enrolled at the time of writing. The expected duration of data collection will be from February 2020 to January 2022, as per our local research ethics board approval (B2018:103). Support for this work has been obtained through the National Institutes of Health (NIH) through the National Institute of Neurological Disorders and Stroke (NINDS) (R03NS114335), funded in January 2020.

**Conclusions:**

With the application of NIRS technology for monitoring of patients with TBI, we expect to be able to outline core relationships between noninvasively measured aspects of cerebral physiology and invasive measures, as well as patient outcomes. Documenting these relationships carries the potential to revolutionize the way we monitor patients with TBI, moving to more noninvasive techniques.

**International Registered Report Identifier (IRRID):**

DERR1-10.2196/18740

## Introduction

Continuous measures of cerebrovascular reactivity by near-infrared spectroscopy (NIRS) provide a convenient and noninvasive method of monitoring cerebral autoregulation in adults with traumatic brain injury (TBI) [1,2]. The concept behind these indices is based on the assessment of the correlation between slow-wave (ie, 0.05 Hz to 0.005 Hz)[3,4] fluctuations in a measure of pulsatile cerebral blood volume (CBV), such as NIRS regional cerebral oxygen saturation (rSO_2_), and a measure of cerebral blood flow driving pressure, such as mean arterial pressure (MAP) or cerebral perfusion pressure (CPP). These indices have been validated in experimental animal models of cerebral autoregulation, with clinical data supporting moderate associations with “gold standard” invasive measures of cerebrovascular reactivity via rough estimates [2,5-7]. However, further investigation into the value of NIRS-based cerebrovascular indices is required.

Several aspects of NIRS cerebrovascular reactivity indices require clarification before widespread clinical application. The first requirement is an in-depth assessment of the time-series relationships between gold standard ICP-derived indices such as pressure reactivity index (PRx) and indices derived from NIRS. Demonstrating strong covariance over time between PRx and NIRS indices would provide confidence in their clinical application for monitoring cerebral autoregulation in adults with TBI. Second, time-series modeling of intracranial pressure (ICP)-derived indices using noninvasively derived NIRS indices could provide a surrogate, noninvasive assessment of PRx. NIRS has never been used for this purpose, although noninvasive modeling of PRx using transcranial Doppler-derived autoregulation indices has been described [8]. Third, the association between cerebral autoregulation during the acute intensive care unit (ICU) phase and long-term follow up has never been assessed using continuous physiologic indices. Those patients with impaired cerebrovascular reactivity during the acute phase of illness may have ongoing dysfunction at 3, 6, and 12 months post-injury. Finally, the association between NIRS-based noninvasive indices of cerebral autoregulation during acute care and long-term follow-up with measures of global functional outcome and patient quality of life have never been assessed. There exists the potential for a direct association between NIRS indices in the acute phase and long-term morbidity/mortality. Furthermore, persistent symptomatology during the long-term phase may be related to ongoing autoregulatory dysfunction, as measured noninvasively through NIRS.

In this paper, we highlight a prospective pilot study that will preliminarily assess all the above questions with NIRS-derived continuous measures of cerebrovascular reactivity during the acute and long-term phases in adults with TBI. We outline the approved protocol for this National Institutes of Health (NIH)-funded study, which has just started recruitment. The specific aims/hypotheses of this project are presented below:

### Aim 1

To compare spatially resolved NIRS-based continuous indices of cerebrovascular reactivity such as cerebral oxygen index (COx), the correlation between rSO_2_ and CPP, and COx-a, the correlation between rSO_2_ and MAP, with “gold-standard” ICP-derived continuous indices such as PRx, pulse amplitude index (PAx), the correlation between pulse amplitude of ICP (AMP) and MAP, and RAC (the correlation (R) between AMP (A) and CPP (C)), using multivariate time-series based assessments of covariance. Demonstrating strong relationships over time between NIRS- and ICP-derived indices will provide confidence in the clinical application of these measures of cerebral autoregulation in adults with TBI.

Hypothesis: NIRS based indices of cerebrovascular reactivity will closely co-vary with gold standard invasively derived ICP indices using high-frequency, high-resolution time-series data.

### Aim 2

To provide models of ICP-based continuous indices (PRx, PAx, and RAC) using noninvasively derived NIRS indices, thus providing the ability to noninvasively measure “gold standard” invasive indices using NIRS in adults with TBI.

Hypothesis: Noninvasive NIRS cerebrovascular reactivity indices can accurately estimate and predict invasive ICP indices using complex time-series modeling techniques.

### Aim 3

To assess cerebrovascular reactivity using noninvasive NIRS continuous indices at 3, 6, and 12 months post-TBI. We expect to demonstrate persistent impairment in vascular reactivity in the long-term phase post-TBI.

Hypothesis: NIRS-based cerebrovascular reactivity will demonstrate some features of impairment during long-term follow-up after moderate/severe TBI.

### Aim 4

To compare cerebrovascular reactivity during the acute ICU phase of illness (using ICP- and NIRS-based continuous indices) and long-term follow up (using NIRS noninvasive continuous indices) at 3, 6, and 12 months post-TBI. We will demonstrate the association between impaired cerebrovascular reactivity during the acute phase, with persistent long-term dysfunction.

Hypothesis: Those with impaired cerebrovascular reactivity during the acute phase of illness will be more likely to display impaired reactivity during long-term follow-up.

### Aim 5

To outline the association between impaired NIRS-based continuous cerebrovascular reactivity during the acute ICU phase of illness with a poor global outcome and patient quality of life at 3, 6, and 12 months. This study will assess global functional outcomes via the Glasgow Outcome Score (GOS) and patient quality of life via the Short-Form 12 Health Survey (SF-12).

Hypothesis: Impaired NIRS based cerebrovascular reactivity during the acute phase of illness will be associated with poor GOS and SF-12 during long-term follow-up.

### Aim 6

To outline the association between impaired NIRS-based cerebrovascular reactivity at 3, 6, and 12 months post-TBI and both poor global outcome and impaired quality of life. This study will compare long-term cerebrovascular reactivity, as assessed via continuous noninvasive NIRS indices, to both GOS and SF-12.

Hypothesis: Impaired NIRS based cerebrovascular reactivity during long-term follow-up will be associated with persistent symptomatology and poor GOS and SF-12 scores.

## Methods

### Patient Population

This study will be conducted at the Health Sciences Center (HSC) in Winnipeg, Manitoba, Canada. Patient recruitment will occur from January 2020 through December 2021. We will be recruiting adults (age 18 years or older) with moderate/severe TBI admitted to the surgical intensive care unit (SICU) and who require invasive ICP monitoring for clinical purposes based on Brain Trauma Foundation (BTF) guidelines [9]. Once ICP monitoring has been initiated for clinical purposes, the patient’s family/legal proxy will be approached for consent for enrollment in the study. Consent will be obtained by a neurosurgical physician assistant or research assistant. Patients/families will have the opportunity to opt-out of the study at any time. Expected recruitment for this pilot prospective observational study will be ~60 to 80 patients, consistent with previously defined sample sizes for high-frequency physiology studies in TBI [10]. Patients will be followed up by the neurosurgery service at 3, 6, and 12 months according to standard post-TBI care at HSC.

All patient demographics, injury characteristics, laboratory, neuroimaging, and outcomes data will be recorded as part of another ongoing prospective moderate/severe TBI database project concurrently running at HSC in Winnipeg (HS20850; H2017:188).

### Physiologic Monitoring

All moderate/severe TBI patients admitted to the SICU at HSC Winnipeg have invasive ICP monitoring initiated as part of standard clinical practice according to the BTF guidelines [9]. As part of standard monitoring within the SICU, patients also receive arterial blood pressure (ABP) monitoring for the duration of their ICU stay. Bifrontal NIRS will be employed continuously for the first 5 days in the ICU, in conjunction with ICP and ABP monitoring.

Follow-up assessment of cerebral autoregulation at 3, 6, and 12 months requires the application of bifrontal NIRS for 30 minutes. Further, in order to derive continuous indices of cerebrovascular reactivity at these follow-up visits, continuous noninvasive ABP will be simultaneously recorded with NIRS with a Finapres ABP system.

### Signal Acquisition

Various signals will be obtained through a combination of invasive and noninvasive methods, with all signals recorded in high-frequency time series using ICM+ software (Cambridge Enterprise Ltd, Cambridge) connected to our SICU monitors [11]. Signals from all of the monitoring devices described below are recorded in time series using this software throughout the recording periods (ie, the first 5 days “acute phase,” 3 months, 6 months, and 12 months follow up). All physiologic signals from monitoring devices within the SICU are recorded and archived as part of a separate prospective signal database study that will be ongoing at HSC (HS20840; H2017:181).

ABP will be obtained through either radial or femoral arterial lines connected to pressure transducers (Baxter Healthcare Corp CardioVascular Group). ICP will be acquired via an intraparenchymal strain gauge probe (Codman ICP MicroSensor; Codman & Shurtleff, Inc). NIRS signals will be recorded bilaterally over the frontal lobes utilizing Covidien INVOS 5100C or 7100 monitoring (Covidien Canada) [Regional cerebral oxygen saturation (rSO_2_) for left and right will be recorded.

Finally, during the follow-up visits at 3, 6, and 12 months, patients will receive simultaneous bifrontal NIRS and continuous noninvasive ABP using the INVOS 5100C or 7100 and Finapres Nano-core systems (FMS, Finapres Medical Systems) [12], respectively. This technique has been previously described by our group [13].

### Signal Processing

All signals will be recorded using digital data transfer or digitized via an A/D converter (DT9803 or 9826; Data Translation), where appropriate, sampled at a frequency of 100 Hertz (Hz) or higher, using ICM+ software. Signal artifacts will be removed manually before further processing or analysis.

Post-acquisition processing will be conducted using ICM+ software. CPP will be calculated using the formula CPP = MAP – ICP. Systolic ABP (ABPs) will be determined by calculating the maximum ABP over a 1.5-second window, updated every second. Similarly, diastolic ABP (ABPd) will be determined by calculating the minimum ABP over a 1.5-second window, updated every second. Pulse amplitude of ICP (AMP) will be determined by calculating the fundamental Fourier amplitude of the ICP pulse waveforms over a 10-second window, updated every 10 seconds.

Ten-second moving averages (updated every 10 seconds to avoid data overlap) will be calculated for all recorded signals: ICP, ABP (which produced MAP), ABPs, AMP, CPP and rSO_2_. Ten-second moving averages will be calculated in order to focus on slow-waves of parent signals, decimating the frequency to the range associated with cerebral autoregulation.

Cerebrovascular reactivity indices will be derived similarly across modalities (ie, ICP and NIRS); an example is provided for PRx: A moving Pearson correlation coefficient will be calculated between ICP and MAP using 30 consecutive 10-second windows (ie, five minutes of data), updated every minute. Details on each index calculation can be found in Table 1. Data for further analysis will be provided in the form of minute-by-minute time trends, output into comma-separated values (CSV) datasets.

### Outcome Assessments

Patient global outcomes will be assessed via GOS at 3, 6, and 12 months post-injury within the standard scheduled clinical follow-up appointments. In addition, patient quality of life will be assessed at these time points via the SF-12 questionnaire, performed in person during the clinic visit. All outcome assessments will be performed by qualified clinic nurses or neurosurgical staff and recorded within the outpatient chart. This form will remain in the neurosurgery outpatient chart, located in GB-1 Health Sciences Center.

**Table 1:** Continuous cerebrovascular reactivity indices to be derived.

Index	Signals Correlated	Signal Averaging (sec)^e^	Pearson Correlation Coefficient Calculation Window (min)^d^	Index Calculation Update Frequency (sec)
PRx	ICP^b^ and MAP^c^	10	5	60
PAx	AMP and MAP	10	5	60
RAC	AMP and CPP^a^	10	5	60
COx	rSO_2_^f^ and CPP	10	5	60
COx_a	rSO_2_ and MAP	10	5	60

^a^CPP: cerebral perfusion pressure

^b^ICP: intracranial pressure

^c^MAP: mean arterial pressure

^d^min: minute

^e^sec: seconds

^f^rSO_2_: regional oxygen saturation

### Statistical Methodology

#### General Statistics

Statistical analysis will be performed utilizing R statistical software (R Foundation for Statistical Computing). Alpha for statistical significance will be set at 0.05, with normality for all continuous variables tested via the Shapiro-Wilks test. Basic descriptive statistics will be performed, with comparisons between groups and variables conducted via t-test, Mann-Whitney-U, chi-square, analysis of variance (ANOVA), Kruskal-Wallis, Friedman, and Joncheere-Terpstra testing, where appropriate. General correlations will be described using Pearson/Spearman coefficients, where applicable.

#### Multi-Variate Co-Variance Analysis

Multivariate testing for inter-index covariance between the ICP and NIRS derived indices will be performed using several methods, including principal component analysis (PCA), agglomerative hierarchal clustering (AHC), partitioning around medoid (PAM) clustering, and K-means cluster analysis (KMCA) [14,15]. A detailed explanation of these statistical techniques is beyond the scope of this section. The strength of clustering in AHC will be confirmed via cophenetic correlation coefficients [16]. KMCA clustering will be validated via the “elbow” methodology.

#### Time Series Techniques

All indices of cerebrovascular reactivity will be output in a minute-by-minute time-series format. Correlation between ICP- and NIRS-based indices will be evaluated in time series using cross-correlation techniques. Using Box-Jenkins time series modeling, the autoregressive integrative moving average (ARIMA) structure of each index time series will be assessed and compared [17,18]. ARIMA model accuracy for each index will be confirmed using autocorrelation function (ACF) and partial autocorrelation function (PACF) plots, augmented Dickey-Fuller (ADF) and Kwiatkowski–Phillips–Schmidt–Shin (KPSS) testing, and the presence of random normally distributed residuals. ARIMA model superiority will be confirmed via ANOVA testing and comparing Akaike information criterion (AIC), Bayesian information criterion (BIC), and log-likelihood (LL) [17-19].

Modeling of ICP-based indices using noninvasive NIRS indices (ie, COx_a) will be performed using a combination of linear mixed-effects (LME) and general linear modeling (GLM), with ARIMA structures embedded within the LME and GLM [17,20]. Model adequacy will be assessed via ACF plots, PACF plots, random, and normally distributed residuals. Model superiority will be confirmed via ANOVA, AIC, BIC, and LL.

#### Association Between Acute and Long-Term Cerebrovascular Reactivity

Comparison between NIRS based indices from the acute and long-term follow-up periods will be conducted, evaluating mean, the integrated area under the curve, and percent time above various thresholds. The analysis will also include comparing PCA, AHC, PMA, and KMCA between the acute and long-term follow up periods.

#### Association With Outcomes

ICP and NIRS indices during the acute phase will be compared with GOS and SF-12 at 3, 6, and 12 months using a variety of linear, proportional odds, and logistic regression techniques. Mean values, the integrated area under the curve, and percent time above various thresholds for the ICP and NIRS indices will be compared to GOS and SF-12 at each timepoint. A similar analysis will occur for the noninvasively derived NIRS indices from follow-up visit recordings, comparing them to GOS and SF-12 results.

### Data Safety/Management

#### Physiologic Data

Physiologic signal data will be managed according to the approved ongoing signal database study, H2017:181. Raw physiologic signal data will be recorded and stored in time-series format by ICM+, then automatically stored on the hard drive of the recording laptops (Windows). These laptops will be password protected, with the ICM+ data files stored in an encrypted password-protected file within the laptop hard drive. ICM+ automatically splits the recording series into 370 MB files in order to reduce data loss in the event of file corruption during the recorded period. These laptops will not be connected to the internet through either hardwire or wireless systems, preventing remote access. All laptops will be stored within the neurosurgery department at HSC behind card-pass and key -protected doors.

All ICM+ datafiles from the recording laptops will be deposited on external hard drives (approximately every 2 weeks), within encrypted password-protected files. These external hard drives will not be connected to the internet, preventing remote access. All external hard drives will remain within the department of neurosurgery, behind locked doors. ICM+ data filenames will consist of unique anonymous codes (000001TBI, 000002TBI, etc), which will be assigned to each patient undergoing data recording/storage on ICM+. The master sheet of patient hospital numbers associated with the unique anonymous codes will be password-encrypted and stored on password-protected computers within the department of neurosurgery. The files are saved in a unique ICM+ file format that can only be opened in the ICM+ software (which will only be available on the laptops recording the data and those of the principal investigator). Furthermore, once the ICM+ files are opened, there is no identifiable patient information stored within, only the anonymous patient identifier and the raw signal information. Thus, there will be no direct way to link this data to any given patient.

#### Demographic and Outcome Data

Demographic and outcome data will be managed according to the approved ongoing database study H2017:188. Demographic and outcome (ie, GOS and SF-12) data will be stored in encrypted password-protected Excel datasheets. Outcome data will be extracted from the neurosurgery outpatient charts, stored in the Section of Neurosurgery, GF-2 Health Sciences Center. This data will be input anonymously into the encrypted Excel datasheets. Files will be stored on password-protected computers within the section of neurosurgery at HSC. All computers will be stored behind card-pass and key-protected doors. Patient names/hospital numbers will be replaced with unique anonymous identifiers (000001TBI, 000002TBI, etc). The master sheet of patient hospital numbers associated with the unique anonymous codes will be password encrypted and stored on password-protected computers within the department of neurosurgery, separate from the Excel database files. The file containing the master sheet of hospital numbers and identifier codes will only be accessible by the principal investigator. Thus, there will be no direct way to link this data to any given patient, ensuring patient confidentiality.

### Patient Safety

There will be no change in patient care. Both the INVOS 5100C/7100 and Finapres Nano-core systems are entirely noninvasive. NIRS monitoring during the first 5 days of ICU stay will not impact clinical care. Any need for transport for neuroimaging will have NIRS discontinued during that time. Follow-up visits will occur at standard intervals within the neurosurgery department for adults with TBI. No extra or additional clinic visits, outside of standard clinical follow up at our institution, will occur. During these visits, the GOS and SF-12 will be assessed. Furthermore, 30 minutes of NIRS and noninvasive ABP recordings will occur during these visits.

### Ethics

Ethical approval has been obtained from the University of Manitoba Research Ethics Board (REB) for both recording and archiving of high-frequency physiologic data in adults with TBI admitted to the ICU or neurosurgical service at HSC, Winnipeg, Manitoba (HS20840; H2017:181). Furthermore, collection of patient demographics, injury characteristics, and outcomes for all adults with moderate/severe TBI admitted to HSC Winnipeg, has also received ethics board approval (HS20850; H2017:188). Approval for prospective recruitment to this study and long-term follow up assessments at 3, 6, and 12 months via NIRS, GOS, and SF-12 has been obtained from the ethics board at the University of Manitoba (HS22191; B2018:103).

### Funding

This project is supported through an R03 project grant from the NIH, through the National Institute of Neurological Disorders and Stroke (R03NS114335).

## Results

Recruitment began at the end of February 2020, with data collection ongoing, with three patients enrolled as of this writing. The expected duration of data collection will be from February 2020 to January 2022, as per our local research ethics board approval (B2018:103). Support for this work has been obtained through the NIH, through the National Institute of Neurological Disorders and Stroke (NINDS) (R03NS114335), funded in January 2020.

## Discussion

Various important aspects of high-resolution physiologic monitoring in TBI will be assessed here, with most assessed for the first time. We hope to prove the ongoing feasibility of bedside ICP- and NIRS-based continuous cerebrovascular reactivity monitoring in adults with moderate/severe TBI in the ICU setting. We expect to show that NIRS-based cerebrovascular reactivity indices will closely co-vary with ICP-based measures such as PRx over time. This data will support the use of NIRS indices as surrogate measures of cerebrovascular reactivity when the derivation of PRx is either not feasible or possible. Furthermore, we expect to demonstrate accurate PRx modeling using NIRS-based measures of cerebrovascular reactivity, potentially providing a noninvasive surrogate for PRx.

Second, we expect to demonstrate the feasibility of the entirely noninvasive technique for cerebrovascular reactivity monitoring in the sub-acute and long-term phases post-TBI. By demonstrating the ability to obtain continuous high-frequency monitoring of cerebrovascular reactivity in a clinic setting, we hope to expand our ability to monitor patients using high temporal resolution techniques into the follow-up phases of care.

Third, assessing the relationship between continuously measured cerebrovascular reactivity during the acute, sub-acute, and long-term phases has never been conducted before this study. We expect to show a strong correlation between vascular dysfunction during the acute phase and persistent dysfunction during follow-up. Furthermore, we expect to demonstrate a link between clinical phenotype in follow-up, and persistent dysfunction in cerebrovascular reactivity. Success here may lead to the replacement of costly and specialized functional MRI techniques in the assessment of not only moderate/severe TBI, but also mild TBI in the subacute and long-term phases post-injury, shifting the focus to clinic/bedside point-of-care monitoring methods.

Fourth, we have been able to publish our technique with NIRS and noninvasive ABP monitoring devices, for the derivation of continuous noninvasive bedside metrics of cerebrovascular reactivity [13]. Our previous work has shown that the technique is feasible and provides continuous long-duration data with limited artifacts. The technique requires only 5 minutes set-up time, most of which is for calibration of the noninvasive ABP device. We expect this exploratory study will allow us to refine the technique and provide a detailed outline of technical steps and procedures. These procedural details will enable other centers and interested parties to be able to employ this bedside technique for both inpatient and outpatient monitoring for various neuropathological states.

Finally, though not a central focus of our work, noninvasive modeling of ICP has long been of interest in the care of patients with TBI. To date, there has been no reliable noninvasive modeling technique for ICP. Our pilot data set from the above-defined protocol will be uniquely positioned to explore some of the time-series relationships between NIRS rSO_2_ and NIRS-derived indices, with ICP. There is the potential that some post-hoc investigations into the NIRS/ICP relationship may provide some useful information for noninvasive modeling of ICP using NIRS.

Through the application of NIRS technology in the monitoring of TBI patients, we expect to be able to outline core relationships between noninvasively measured aspects of cerebral physiology and both invasive measures, as well as patient outcomes. Documenting these relationships carries the potential to revolutionize the way we monitor TBI patients, moving to more noninvasive techniques.

## Abbreviations

ABP: arterial blood pressure
ABPd: diastolic arterial blood pressure
ABPs: systolic arterial blood pressure
ACF: autocorrelation function
ADF: augmented Dickey-Fuller
AHC: agglomerative hierarchal clustering
AIC: Akaike information criterion
AMP: pulse amplitude
ANOVA: analysis of variance
ARIMA: autoregressive integrative moving average
BIC: Bayesian information criterion
BTF: Brain Trauma Foundation
CBV: cerebral blood volume
CPB: cerebral perfusion pressure
CSV: comma-separated values
GLM: general linear modeling
GOS: Glasgow Outcome Score
ICP: intracranial pressure
ICU: intensive care unit
KMCA: K-means cluster analysis
KPSS: Kwiatkowski–Phillips–Schmidt–Shin
LL: log-likelihood
LME: linear mixed-effects
MAP: mean arterial pressure
NIRS: near-infrared spectroscopy
PACF: partial autocorrelation function
PAM: partitioning around medoid
PCA: principal component analysis
rSO_2_: regional cerebral oxygen saturation
SF-12: Short-Form 12 Health Survey
SICU: surgical intensive care unit
TBI: traumatic brain injury
